# The Power Is in the Word—Do Laypeople Interpret Descriptors of Dog Emotional States Correctly?

**DOI:** 10.3390/ani13193009

**Published:** 2023-09-25

**Authors:** Carmen Heritier, Stefanie Riemer, Robert Gaschler

**Affiliations:** 1 Department of Psychology, FernUniversität Hagen, Universitätsstraße 47, 58097 Hagen, Germany; 2 Comparative Cognition, Messerli Research Institute, University of Veterinary Medicine Vienna, Medical University of Vienna, University of Vienna, 1210 Vienna, Austria; riemer.stefanie@gmail.com

**Keywords:** dog behaviour, emotional states, qualitative behaviour assessment, QBA, descriptors, laypersons, veterinary situation, shelter situation

## Abstract

**Simple Summary:**

Dogs are part of our everyday lives. It is therefore important that descriptors and definitions used to label their emotional states are understood correctly. Are laypersons able to interpret basic dog behaviour and emotions correctly in order to prevent dangerous situations? This study compared whether descriptors, such as “fearful”, could be matched to their correct definitions, e.g., “dog may try to flee, hide or freeze; ears back”, by laypersons. To this end, two sets of descriptors—one for veterinary situations and one for shelter situations—were used. Matching was substantially above chance; nonetheless, the mean proportion of correct responses was only 50% (SD ± 16.6%) for the veterinary QBA set and 33% (SD ± 14.3%) for the shelter QBA set. Emotional terms describing dog behaviour need to be clearly defined to avoid misinterpretations.

**Abstract:**

A basic understanding of dog behaviour and emotion is relevant not only for professionals, such as veterinary personnel or dog trainers, but also for dog owners and for people with little contact with dogs. Information about dog behaviour and emotions is mostly conveyed verbally. This study explores whether definitions of dog behaviour and emotion are understood in such a way that they can be allocated to a descriptor (i.e., a label such as “fearful”), even by people with low background knowledge. If people can match descriptors to definitions, this suggests that the definitions are distinct enough and elicit mental representations of behaviour that can fit the label. Good agreement on the definitions is a prerequisite for the validity of the descriptors used; however, no study to date has tested this. A sample of 236 adults was asked to match descriptors of Qualitative Behaviour Assessment (QBA) for veterinary and shelter situations to their correct definitions, e.g., the descriptor “fearful” to its definition “dog may try to flee, hide or freeze; ears back”. Matching was substantially above chance; nonetheless, the mean proportion of correct responses was only 50% (SD ± 16.6%) for the veterinary QBA set and 33% (SD ± 14.3%) for the shelter QBA set. Performance in the matching task was positively correlated with measures of experience with dogs. Taken together, the results suggest that descriptor–definition pairs used to describe dog behaviour need to be clearly defined to avoid misinterpretations when teaching laypeople how to interpret canine behaviour.

## 1. Introduction

The question of whether humans are capable of judging other species’ emotions has occupied scientists ever since Darwin’s The Expression of the Emotions in Man and Animals [1]. Darwin’s theory that the expression of emotional states in animals and humans is inherited has been widely discussed [1,2,3]. A part of his revolutionary idea was that not only is the expression of emotional states inherited but also their recognition [1]. Darwin compared the facial expressions of humans and, amongst other species, dogs. As mammals share the same basic emotional neuroanatomy [4], Darwin’s ideas do not seem to be too far-fetched. Nonetheless, there is evidence that facial emotional expressions are species-specific [5] and interpretation of these cues by untrained people can be difficult. "Puppy dog eyes" (raising the inner brow area in dogs), for example, were thought to have a communicative function in dog–human interactions, but their appearance seems to be connected with eye movement [6]. Modern behavioural research follows up on some of Darwin’s ideas by assessing people’s ability to identify emotional states in dogs. This identification process would be especially important when it comes to recognising negative emotions in dogs that can lead to potentially dangerous situations. 

There is evidence that verbal descriptions are important to communicate and learn about dog behaviour. A description provided to another person might convey an interpretation and highlight relevant aspects of dog behaviour, thus directing the listener’s attention and suggesting a meaning of canine behaviour. Verbal descriptions are widely used in teaching about dog behaviour [7,8,9]. From books targeting a preschool audience [10] to primary school teaching materials provided by the German Federal Ministry of Food and Agriculture [11], there is explicit teaching for people of different ages on how to interpret dog behaviour. This seems especially important as human misinterpretation of canine communication is the number one reason for dog bites [12]. A recent review of dog bite prevention strategies indicated that while the education of children was not effective, intensive adult-directed education may aid in reducing dog bites [13]. Studies on educating owners or the general public on dog behaviour are scarce; nonetheless, the importance of this education is stressed and further studies are recommended [14]. 

While classic research on behavioural indicators of affective states in dogs mainly focuses on quantitative measures, such as ear position and amount of lip-licking or tail movement [15,16], Qualitative Behaviour Assessment (QBA) is an approach that focuses on the animal as a whole [17] and integrates nuances of the shown behaviour in its assessment. As observers rate the expressive quality of a shown behaviour—listed as descriptors, e.g., “nervous”—on a visual analogue scale, it is easily applicable in live or video assessments. 

Agreement of observer ratings varies from study to study and the effects of the observers’ experience have been investigated. Although a 2021 QBA study using free-choice profiling indicated above-chance consensus without training [18], both raters’ expertise and prior training on the use of the tool improved reliability levels [19,20]. 

Initially, QBA was based on free-choice profiling in which experts freely generate and score their own list of descriptors [17]. However, recent QBA studies mostly use pre-defined descriptors to be rated by observers. This is perceived as more suitable for assessment in real-life situations, such as on a farm [19,21]. Moreover, it facilitates the use of QBA for comparative assessments of animal welfare [21,22,23]. 

Different studies, however, have been found to differ in their definitions of the same terms [24]. Therefore, it is paramount to train observers on how to apply these terms [25]. No doubt, clearly defined descriptors are a prerequisite for reliable assessment in QBA. Papers on the quality of the definitions and their interpretation do exist [19,26] and demonstrate the necessity of aligning the scoring styles of the observers, but it has not been investigated how well descriptors and definitions are understood and can be matched by the raters. 

The discrepancy between “labelling” and “understanding” has already been touched upon in a 2009 study [27] in which 60 observers were asked to label the behaviour of 9 dogs from videos. Although the study did not use the QBA method, observers were expected to classify the behaviour of the dogs with adjectives, such as “aggressive” or “fearful”. The different backgrounds of the observers, whether they worked with dogs professionally or not, had no significant effect on the correct allocation of the adjectives. The findings suggest that humans tend to use a holistic approach when observing dogs, but due to the methodology applied in this study, distinguishing between mislabelling or misinterpreting dog behaviour is not possible [27]. This issue served as a starting point for our investigation.

We tested how well QBA descriptors representing dog behaviour could be matched to their definitions by laypersons, i.e., to what degree definitions from one set of published descriptors and a novel set can be intuitively understood, and how it is affected by participants’ dog-related experience.

In other fields, such as product evaluation, assessing the matching of descriptors and definitions is a common approach. In wine tasting, for instance, descriptors such as “fresh” or “deep” [28] are used to describe a wine’s taste, but the definitions of these descriptors often remain unknown. In a 2021 study, wine tasters were asked to define given descriptors [29]. With tasters listing contradicting definitions, the need for clarification in this area became apparent [29]. Moreover, the descriptors’ definitions do have an influence, for example, on a customer’s choice of which wine to buy [30]. There is even research on how descriptive food names can bias sensory perception [31]. 

Verbal knowledge is relevant for communication and might furthermore dominate memory traces [32]. Given that people vary strongly in how much contact they have with dogs, it is relevant to explore whether high-quality verbal knowledge about dogs is mostly a characteristic of persons with a lot of experience with dogs, or—as would fit the educational aim of books for children and parents about dogs—whether it is found in many people, including those with relatively little contact.

Previous studies on the role of experience in assessing animals’ affective states, using QBA, have yielded mixed results. An earlier study on QBA found that an observer’s level of experience with the observed species, or even with other species, can influence the observer rating [33]. Another study found that the probability of selecting the descriptor “fearful” to describe dogs showing signs of fear increased with experience [34]. A study on laypeople’s and dog owners’ recognition of facial expressions in both dogs and humans listed experience with dogs as one contributing factor, with empathy and personality influencing the rating as well [35]. A 2021 study even suggests crowdsourcing by naïve observers as a useful tool to assess dog behaviours, with reliability being achieved by as few as 10 raters [36].

### 1.1. Qualitative Behaviour Assessment (QBA)

QBA is a method used to describe the likely emotional states of nonhuman animals (e.g., farm animals [37,38,39,40,41,41,42], zoo animals [43,44,45], and domestic dogs [23,46,47]), following a “whole” animal approach [17]. Rather than just measuring gross behaviours, i.e., *what* the animal is doing, QBA focuses on the expressive style with which an animal is performing a behaviour [48], i.e., the animal’s demeanour and the question of *how* the animal is doing what it is doing [19]. In order to measure the “how” of an animal’s behaviour, QBA relies on observer ratings that target complex behaviour patterns rather than single features. Therefore, instead of counting how many times a specific behaviour is shown, as would be the case in traditional behaviour assessment, observers rate the demeanour of an animal over a short period of time. This is carried out by allocating a value to individual descriptors, such as “excited”, on a visual analogue scale [48].

In QBA, observers are either asked to generate their own descriptive terms (Free-Choice profiling) or are provided with a pre-defined list of descriptors based on literature research and expert choice [19]. 

#### 1.1.1. Free-Choice Profiling 

In order to rate an animal’s behaviour via Free-Choice Profiling (FCP), an observer has to first create a list of terms for the shown behaviour [20]. This implies that the terms used by different observers might be unique or differ greatly from each other. All observers rate the same animals and behaviours, either live or based on video footage. Generalised Procrustes Analysis (GPA) is used to identify common patterns in the observer’s terms [20]. A Principal Component Analysis simplifies the data into main dimensions [20]. 

#### 1.1.2. Fixed Lists 

An alternative approach is the use of fixed lists of descriptors and their corresponding definitions, which are pre-defined by the experimenters. Some studies use descriptors and definitions that are universally agreed upon by leading scientists or certified—such as the European “Animal Welfare Project” (AWIN) for donkeys, goats, horses, and sheep [26,49,50,51]. Other descriptor–definition pairs are even part of registered trademark protocol—such as the Welfare Quality^®^ protocols for cattle, poultry, and pigs [4]. Other studies use their own descriptors and definitions and justify why they deviate from the terms regularly used in the specific species [33,52].

The advantage of using fixed lists of descriptors is that they can be used for relatively standardised animal welfare assessment and reduction in observer bias [53,54]. When compiling fixed lists of QBA descriptors, the first step is to review the relevant literature in order to identify suitable descriptors, such as “stressed” or “fearful”. Reflecting the complexity of animal behaviour, it is on purpose that terms in the definitions are partially overlapping [19]. In the second step, expert opinion is consulted about which descriptors to select. The information on this procedure in the method section of several papers varies from very detailed [19,23,33] to rather roughly sketched or not mentioned at all [46]. Training the observers is mentioned as a very important third step when using pre-defined lists of descriptors. This is meant to ensure inter-rater reliability and was tested for 10 or more observers [21,33]. While ratings from untrained persons may be reliable as shown for QBA in pigs [17,55] and cattle [40], training observers have been shown to reduce observer bias [53]. With untrained observers, external validity may be limited [56]. 

To achieve high inter-observer reliability, it is paramount that the descriptors are clearly defined. A 2019 study on QBA in dogs [19] generated the definitions of the descriptors on the basis of expert feedback, which included questions such as ease of understanding and ambiguity of the definition. However, it has never been assessed how intuitive such definitions are and whether observers might already differ in their interpretations of the descriptors, independent of the animal’s behaviour. Good agreement on the terms used is a pre-requisite for the validity of the descriptors, but to date, no study has tested this. 

Different QBA studies vary in how the terms on the list are presented. In some studies, contrasting terms are presented adjacent to each other [33]; in others, the order is randomised [57]. Some studies do not mention the chosen order [19,21,33] and do not explain the reason behind it [22,23,24]. In the current study, we assessed the ability of non-experts to correctly allocate the definitions to their corresponding QBA descriptors. We used two QBA sets: one previously published QBA set describing dog behaviour in a shelter [23] and one QBA set for dogs in a veterinary setting. The QBA set for dogs in veterinary situations was compiled for this study, as there is no QBA set available for this specific purpose. Our aim in testing two QBA sets was to be able to compare the outcome of the veterinary QBA set to that of an established QBA protocol. We assumed that if the veterinary QBA descriptor set gained the same or more agreement between descriptor and definition pairs, this could be used as a reference. 

We investigated how accurately participants could match descriptors, e.g., “nervous”, with their corresponding definitions, e.g., “unsure, shy, looking around, vigilant”. Matching descriptors and definitions can serve two purposes. One is a quality check for a given QBA list which provides the basis for using the list in further studies. If people can match definitions to descriptors, this suggests that the definitions are distinct enough and elicit mental representations of behaviour that can fit the label. Second, we investigated the effect of participants’ prior experience with dogs on their ability to allocate the descriptors and definitions correctly. 

## 2. Materials and Methods

### 2.1. Compilation of Descriptors

We used two different QBA lists. One was developed by the authors for describing dog’s behaviour in a veterinary setting (unpublished). The other one was a translated version of the list from a QBA study on shelter dogs [23]. Using two QBA lists allowed us to explore the generalizability of the findings. 

#### 2.1.1. Descriptors for Veterinary Settings 

Twelve descriptors of dog behaviours were used (in German; English translation is provided in Table 1. The original German terms are listed in Appendix A). The selection of the descriptors took place in several stages of refinement. Five experts on dog behaviour—three veterinarians specialising in behavioural medicine, working at a German University, and two experienced dog trainers—were asked to suggest behavioural descriptors after seeing three videos of dogs during a standardised veterinary examination. These videos were part of a previous study conducted at the Vetmeduni Vienna, Austria [58]. The descriptors mentioned by the experts were then grouped, as several descriptors were used to describe nuances of the same behaviour and re-evaluated by the expert group in a second round. In a third round, the expert group formulated definitions (describing specific behaviours) for each descriptor based on a literature search in the field of QBA [19,23,48]. These definitions were then refined by the authors.

#### 2.1.2. Descriptors for Dogs in Shelters 

Twenty descriptors from a study on QBA for use with dogs in a shelter environment [23] were also included in the current study. As the present study was performed in Germany, the original Norwegian descriptors and their definitions from [23] had to be translated into German (see Appendix A). The translation was based on the published English version. Whenever the English descriptor or parts of its definition had several equivalents in German, the Norwegian original was consulted to facilitate translation. 

### 2.2. Questionnaire Design

The online survey was set up in Unipark [59]. In order to describe the sample, we asked for participants’ gender and age range. Furthermore, we enquired about the amount of contact with dogs estimated in days per year for the last 3 years (on average), knowledge about dogs, experience with dogs, origin of experience with dogs, and professional contact with dogs (Appendix B). In the next step, participants were asked to allocate each of the 12 QBA descriptors for a veterinary setting to the definitions. The participants were shown a definition—as seen in the right column of table [1]—and had to allocate the matching descriptor from a drop-down menu. Both descriptors and definitions were randomised individually for each participant.

Afterwards, the participants performed the same task on the 20 descriptors and definitions from the QBA study on shelter dogs [23]. The questionnaire ended with questions on participants’ seriousness, stressing the importance of correct participation and thereby ensuring the validity of data. Participants could be excluded when ticking the box that they had not taken participation seriously, which no participant indicated.

### 2.3. Participants

Data collection took place via the virtual lab of the psychology department of FernUniversität in Hagen, Germany. This lab is openly accessible but mostly attracts students of psychology as they can earn credit points for participating in surveys as part of their study programme. A total of *N* = 270 people took part in the survey, with *N* = 261 completing it. Data of *N* = 236 could be used. *N* = 25 subjects had to be removed from the analysis because the time to complete the questionnaire was so short (t < 5 min) that reading the questions would not have been possible, they had spent more than six hours working on the questionnaire, or admitted to not taking the task seriously.

### 2.4. Statistical Analysis

The results of the two sets of QBA descriptors were analysed independently of each other. Descriptive statistics were used to analyse the proportion of correct allocations for each descriptor. More detailed analyses followed showing which descriptors were used instead of the correct one for both QBA lists. 

In order to understand the effects on the proportion of correct allocations, several items were further investigated. The items described as knowledge about dogs and experience and contact with dogs in days were correlated with each other using Spearman’s rank correlation coefficient. An experience score was established following a z-standardisation by taking the mean of the three z-standardised variables. The score consisted of the variables “direct contact to dogs”, “expertise” and “experience”. This experience score was then correlated with the percentage of correct allocations using Spearman’s rank correlation tests.

## 3. Results

We begin by reporting on the matching of descriptors to definitions for the veterinary QBA list developed in the current study before we provide corresponding data for the shelter QBA list by Stubsjøen et al. [23].

### 3.1. QBA during a Veterinary Examination 

#### 3.1.1. Correct Assignment of Descriptors and Definitions

The mean rate of correct matching of descriptors to definitions was *M* = 50% (SD ± 16.6%) for the list of veterinary QBA descriptors. This is considerably higher than the guessing probability of 8.33% (=1/12; assuming independence of descriptors; t(235) = 38.73, *p* < 0.001). Only *n* = 4 persons out of *N* = 236 participants scored 8.33% or less in correct allocations. When assuming that the sample was guessing, one would expect *N*/2 = 118 participants with a result at or below the guessing baseline.

**Figure 1:**
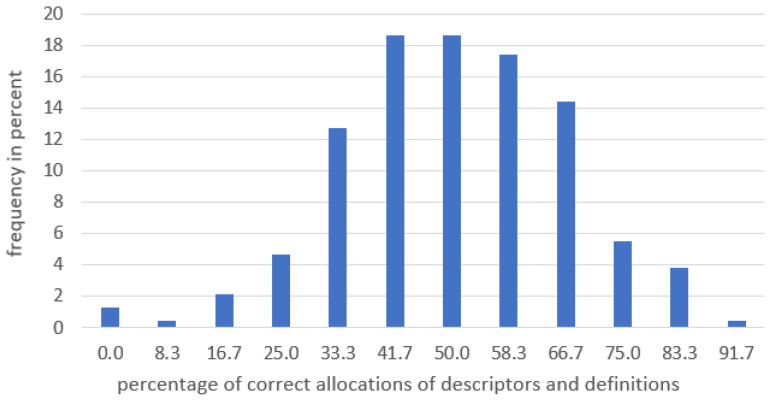
Distribution of correct allocations between descriptor and definition.

The distribution of correct allocations ranged from 0% to 91.76% (Figure 1). Most commonly, the proportion of correct allocations was between 41% and 58%. Only 8.5 per cent of the participants allocated less than or up to twenty-five per cent of descriptors correctly. 

#### 3.1.2. Role of Previous Knowledge of and Contact with Dogs

The questions evaluating knowledge, experience, and contact with dogs in days were highly correlated with each other. Knowledge about dogs (with Spearman’s Rho = 0.616, *p* < 0.001) and experience with dogs (Rho = 0.666, *p* < 0.001) were positively correlated with contact with dogs in days. Furthermore, knowledge about dogs and experience with dogs were also highly correlated (Rho = 0.772, *p* < 0.001).

The three items measured experience with high reliability according to Cronbach’s alpha (α = 0.84), which was used as a measure of internal consistency. Accordingly, an experience score was computed by Z-standardizing and averaging these three items. 

**Figure 2:**
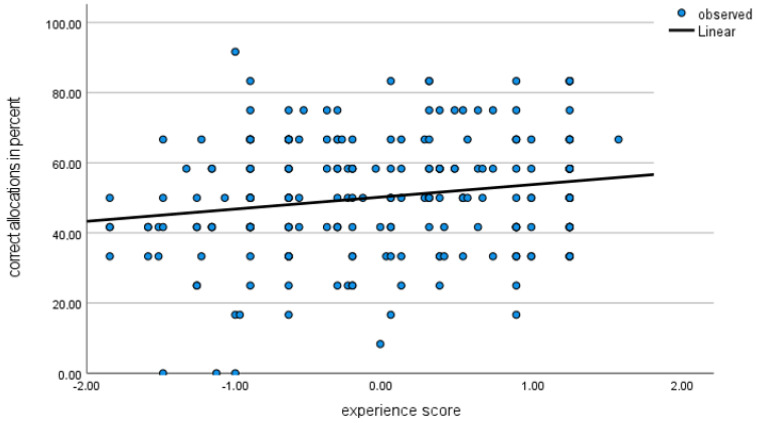
Scatter plot illustrating the percentage of correct allocations (y-axis) against the standardised experience score (x-axis).

As shown in Figure 2, the score could then be used to assess the extent to which classification accuracy was correlated with experience, Rho(234) = 0.162, *p* = 0.012.

#### 3.1.3. Detailed Analyses of Correct Assignment of Descriptors and Definitions

In the next step, we explored which descriptors were characterized by high matching accuracy and which received low matching accuracy. As shown in Table 2, high matching scores (≥66.666) were obtained for “relaxed”, “aggressive”, “sociable”, “curious”, and “fearful”. Moderate scores were obtained for “tense”, “submissive”, and “stressed”. Low accuracy (≤33.333%) was found for “nervous”, “lively”, “restless”, and “excited”.

Besides analysing the proportion of correct allocations, the specific errors in matching descriptors and definitions can inform about typical misunderstandings shown by laypeople. Table 3 shows which descriptors were allocated to which definitions by the participants. For the definition of the descriptor “aggressive”, 77% of participants chose the correct descriptor, with 7% choosing “tense”, 4% choosing “fearful”, 1% each choosing “lively” and “nervous, 7% choosing “stressed”, and 1% choosing “excited”. 

Based on the above results, we decided to refine the descriptor list to avoid confusion and save rating time in future studies. Four descriptors showed only low correspondence with their definitions. Those descriptors were “excited” (17%), “restless” (22%), “lively” (22%), and “nervous” (30%). The reason for this low correspondence might be that the definitions and the descriptors were perceived as rather similar, with “excited” and “lively” expressing an emotional state of positive valence and “restless” and “nervous” expressing negative valence. The descriptor “excited” was allocated correctly by only 17% of subjects, with 56% of them matching it with “lively” and 19% with “sociable”.

As a result, we decided to drop the terms “excited” and “restless” from the list for future studies and keep the descriptors “lively” and “nervous”. This leads to the following descriptor–definition list, as shown in Table 4. 

### 3.2. QBA in Shelter Dogs

#### Correct Assignment of Descriptors and Definitions

**Figure 3:**
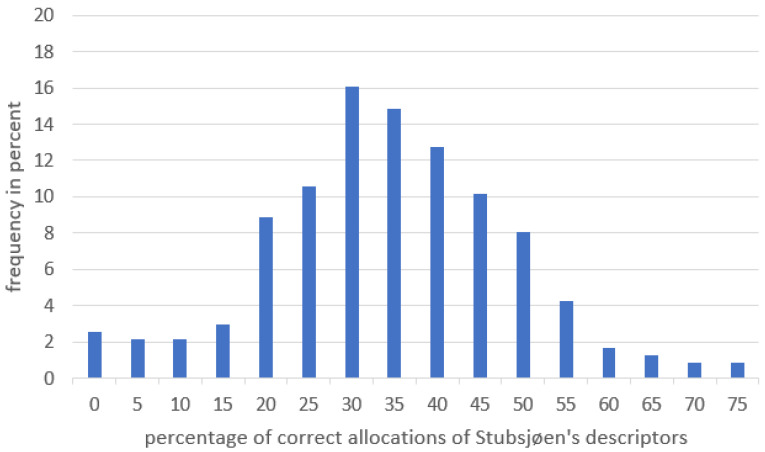
Distribution of correct allocations between descriptor and definition for the list by Stubsjøen et al. [23].

**Figure 4:**
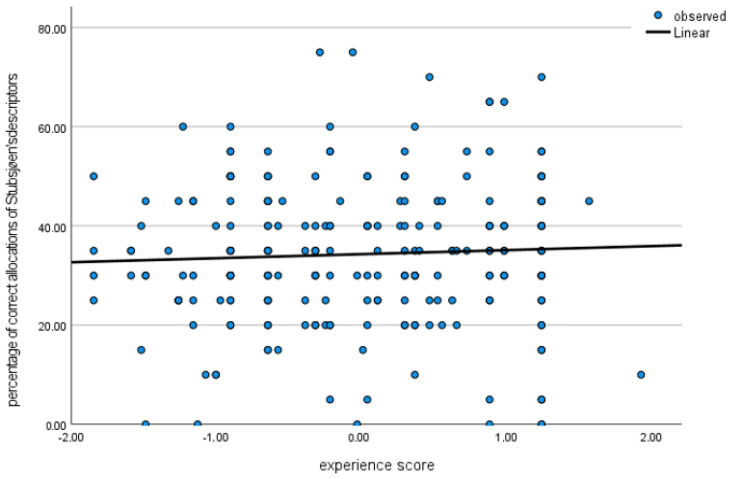
Scatter plot illustrating the percentage of correct allocations (y-axis) against the standardised experience score (x-axis) for the descriptors of Stubsjøen et al. [23].

The mean rate of correct matching of descriptors to definitions was *M* = 33% (SD ± 14.3%) for the descriptors of the Stubsjøen et al. [23] list. The distribution of correct allocations ranged from 0% to 75% (Figure 3)—the latter implying that 15 out of 20 descriptors were allocated correctly to their definitions. There was no correlation between the experience score and classification accuracy, as tested by Spearman’s Rank Rho(234) = 0.073, *p* = 0.267 (Figure 4). 

For the shelter dog descriptors [23], “playful”, “depressed”, “sociable”, and “aggressive” showed the best correspondence between descriptor and definition. “Relaxed”, “trustful”, “energetic”, “curious”, and “indifferent” showed moderate correspondence. A total of 11 descriptors showed a very low correspondence of under 30% (Table 5).

### 3.3. Correlation between Shelter and Veterinary QBA Sets

The proportion of correctly matched descriptors in the Stubsjøen et al. list and the list presented in Section 3.1.1 showed a correlation of Spearman’s Rank Rho(234) = 0.362, *p* < 0.001. Participants who performed well on the matching task with one list tended to perform comparatively well on the other list.

## 4. Discussion

The current study explored how well laypersons could match descriptors and definitions of two Qualitative Behaviour Assessment (QBA) sets in dogs. Since our purpose was to assess how well definitions are understood “intuitively” by laypersons, the subjects received no prior training. This deviates from the procedure where QBA is used for emotional assessments. In most studies, prior training for observers forms an integral part of QBA with fixed descriptor lists [39,44,60]. Given the relevance of verbal knowledge and categories in transmitting knowledge about dog behaviour and emotion, the manuscript contributes by showing that laypersons can deal with verbal descriptors rather consistently—even if they lack experience with dogs. This can, for instance, provide a starting point for studies testing how pictures and text can be combined efficiently to assist in the knowledge acquisition of laypersons (cf. [61]). Of course, such approaches should not replace video-based training and live training. Yet they might accompany the latter. They can also provide an additional low-threshold and easily available source for spreading knowledge about dog behaviour and emotions. 

The correct allocation of descriptor–definition pairs was above chance level, with participants correctly matching on average 50% of descriptor–definition pairs of the veterinary situation QBA list and 33% of the shelter situation QBA list. There was a low but significant correlation with experience with dogs, but only for the veterinary QBA set. Based on the analysis of the matching mistakes, we concluded that shortening the veterinary QBA tool from 12 to 10 descriptors will likely improve clarity. The length of the shelter QBA tool with 20 descriptors could possibly explain why the proportion of correct matches for the shelter QBA descriptors was lower than for the veterinary QBA descriptors. While partially overlapping descriptors are inherent in the purpose of QBA and seem to be needed in order to fully grasp the emotional state of the animal in question [17,56], a simplified version with 10 descriptors (cf. Table 4), with less overlap, might prove helpful for quick evaluations. These quick evaluations, in turn, might not be as exact as the original approach with more descriptors, but they can be useful when applied in real-life situations under time constraints. 

### 4.1. Comparison of Correct Allocations between the Datasets from Veterinary and Shelter QBA

The difference in correct allocations of descriptor–definition pairs between the shelter QBA list [25] and the veterinary QBA list could have several reasons. As more time is needed to match 20 descriptors in comparison to 12 descriptors, fatigue could contribute to lower success rates for the shelter QBA. Furthermore, the shelter QBA descriptors were always presented after the veterinary situation QBA descriptors, potentially increasing the contribution of fatigue. Fatigue or proactive interference is not an explanation for low performance on individual descriptor–definition allocations, as the order was randomised between participants.

At the individual level, certain descriptor–definition pairs were matched correctly more often than others. The highest correct allocation rate for the veterinary QBA set was achieved for the descriptors “relaxed”, “aggressive”, “sociable”, “curious”, and “fearful”. These descriptors seem to possess very distinct qualities that allow for easy identification. The high matching rate of “relaxed” and “aggressive” could be explained by the antonymic description “not hectic” for “relaxed” and the direct quotation of “aggression” for “aggressive”. Note that in QBA, words from the same word stem are often used in the definition of descriptors; therefore, we also used this approach in our novel veterinary set. While the behaviour shown by a dog categorised as “fearful” or “aggressive” could potentially be assessed wrongly by observers, the concise definitions prevented a high amount of mismatches. Only 4% of participants mistook “fearful” for “aggressive” and 0% mistook “aggressive” for “fearful” (cf. Table 3).

Although “affiliative behaviour”—a rather technical, scientific term—was included in the definition of “sociable”, mentioning “contact/interaction” might have enhanced correct matches. The verb “seek” in “seeking contact”, however, implies that the activity was initiated by the dog itself. The inclusion of “seeking contact” in the definition of “sociable” might have led to 15% of participants mistaking “sociable” for “curious”. As the behaviours—apart from the words describing them (such as approaching and making contact)—can appear very similar, this might be the reason behind those mismatches. 

For the shelter QBA set, only three descriptors achieved allocation rates of over 60%. Those were “sociable”, “aggressive”, and “playful”. The definition of “sociable” is very similar to that of the veterinary situation QBA set, with “seeking contact/interaction, friendly, positive interaction with other dogs” (cf. Appendix B), leaving only little room for interpretation. As in the veterinary QBA set, the descriptor “aggressive” was defined by stating “offensive or defensive aggression”, already hinting at the descriptor. In the definition of the descriptor “playful”, the word stem of “play” is mentioned twice; therefore, attentive participants would have found it easy to correctly allocate this pair. 

Correct allocations of the descriptors “nervous”, “frustrated”, “stressed”, and “restless” ranged from 13% to 26%. This low success rate could be explained by the similarity of the behaviours behind those descriptors—all of them seem to share a negative valence and stress is inherent in nervousness, frustration, and restlessness. As in the veterinary QBA set where we decided to reduce the number of descriptors from 12 to 10 to exclude descriptors that are similar and easily confused, this hints at fewer, more distinct descriptors being preferable. 

Apart from the linguistic aspect of allocating definition and descriptor pairs correctly, it is interesting to note that the term “aggressive” ranked very high in both QBA sets, with 77% and 64% correct allocations, respectively. A reason for this could be the perceived importance of correctly allocating this descriptor, as aggressive behaviour could pose a real threat to the participant, drawing more attention to this definition, as well as the word “aggression” in the definition.

### 4.2. Experience

In the current study, overall, the effect of experience was low for the veterinary QBA set and nonsignificant for the shelter QBA set. Previous studies yielded mixed results with regard to the impact of prior experience on assessing behaviour and emotions. 

The perception of emotion in other humans has been shown to be influenced by an individual’s cultural background and especially by childhood experience [62,63,64]. However, in studies investigating interspecific emotion perception, little evidence has been found for the modulating effect of experience on observers’ ability to infer likely emotions in dogs [27,65]. Neural networks of emotion processing may be applied flexibly to both interspecific and intraspecific [34,66] contexts. 

In regard to QBA, earlier studies found that the observers’ level of experience with the observed species, or even with other species, can influence the observer rating [33]. In the current study, we assessed whether observers of different experience levels differ in their ability to match descriptions to their QBA descriptors, i.e., whether the descriptors evoke correct mental representations. The study indicates that an individual’s experience with dogs has only limited impact on this individual’s capability to correctly match descriptor and definition pairs of dog emotional states. A low correlation between the proportion of correct allocations and experience score was found only for the veterinary QBA set.

The findings on the correct allocation of descriptor–definition pairs and the role of experience, however, could be limited to the linguistic understanding of descriptors and definitions and might not necessarily reflect a correct assessment of a dog’s emotional state when experienced live or seen on video. A future study is planned to assess the correspondence with video ratings of dogs.

### 4.3. Verbal Descriptions

Working on and improving verbal descriptions might provide a basis to address the even larger complexity inherent in video material or direct observation. The complexity of assessing animal behaviour also lies within the fact that animals are being assessed by humans who might or might not share different experiences and beliefs. A study from 2014 asked, “Can we believe what we score, if we score what we believe?” [53]. It concluded that observer bias is a more important problem in animal behaviour research than expected. An earlier review on animal behaviour research [54] stated that at least either inter-rater reliability should be assessed or blinded coding of several conditions should be used to decrease observer bias. Assessing the consistency of different raters in categorizing verbal descriptions can constrain sources of inconsistency when working with direct observation or video material. In quantitative coding, it is sufficient that one person does the coding if reliability with a second coder can be demonstrated based on a subset of the videos [67]. For qualitative ratings, at least 10 people are suggested to ensure inter-rater reliability based on measures of correlations between individuals [36,68]. 

Apart from the number of raters, the language used for the descriptors and definitions is very important. The Sapir–Whorf hypothesis, also described as the linguistic relativity hypothesis [69], states that language has a strong impact on our perception of the world. Following this approach, the importance of the way definitions and descriptors in QBA are phrased becomes apparent. More recent research takes into account that language emotionality influences an individual’s perception [70]. It is therefore essential to further explore the linguistic power behind descriptor and definition pairs as one constituent to the validity of Qualitative Behaviour Assessment. Further QBA studies could perform an a priori check of their descriptor–definition pairs in order to identify whether there may be any misunderstandings or misconceptions. 

With regard to future dog bite prevention programmes, it could be useful to do the same. Clarifications of what it means when an educator talks about a fearful dog and what “fearful” can look like, could enhance education. Supporting pictures or videos seem inevitable in order to enhance learners’ ability to assess the potential danger associated with certain emotional states or behaviours a dog shows. 

### 4.4. Practical Applications

The current study indicated that a smaller number of descriptors may improve observers’ agreement on and/or comprehension of the descriptor definitions. For valid animal welfare assessments using QBA, several observers are needed [36,68,71], and it is unquestionable that more descriptor–definition pairs provide a more detailed representation of a dog’s behaviour. Nonetheless, we suggest that alternative approaches adapted from QBA may be of value in practical settings where the necessary number of observers and QBA descriptors cannot be achieved, such as monitoring dog welfare in a veterinary setting. Similarly, a recent study on horse welfare [72] has shown that as little as seven descriptors were useful to assess the horses’ emotional expressivity in a racing environment. Prior training of veterinary staff on a smaller number of clearly defined descriptors allows their use in situations where circumstances, e.g., time constraints, do not allow for extensive use. Thus, dogs’ emotional states could be assessed simultaneously with procedures taking place, and counter-measures could be taken when necessary, resulting in better welfare of the canine patients, as well as their guardians and veterinary staff [73].

## 5. Conclusions

QBA is a valuable tool for assessing animal welfare by focusing on the (likely) emotional experience of the animal, thereby stressing the expressive quality of behaviour and underlining nuances in this behaviour. Adopting similar approaches may also have value in settings such as veterinary offices to make quick assessments of dogs’ emotional states, as well as when educating laypeople on dog emotional expressions. The current research focused on an earlier step, namely, the question of whether the descriptors used can be understood intuitively and whether laypeople can match them to their expert-derived definitions. When nuances in behaviour are at stake, it is paramount to clarify whether descriptors and definitions are understood the way researchers think they are. Our study highlighted the importance of prior training of raters and of clear definitions when a fixed-list approach is used.

Previous studies have explored the impact of different methods of educating children and adults about dog behaviour, mostly in order to prevent harmful incidents. However, no study so far has assessed how well the terminology used to describe relevant behaviours is understood by the learners. This study demonstrates the significance of the wording used to avoid ambiguities, thus providing new insights to improve education about dog behaviour.

## Figures and Tables

**Table 1:** The list of descriptors and definitions in English.

Terms	Definitions
Aggressive	Shows signs and posture of defensive or offensive aggression (staring, growling, showing teeth, snapping into the air or towards people, etc.)
Tense	Muscular tension; stiff body, may appear frozen
Curious	Interested in surroundings, looking at or approaching people or objects on its own, neutral posture, ears neutral or forward
Fearful	Dog may try to flee, hide, or freeze; ears back
Lively	Moving around with the legs or body but not apparently in an attempt to avoid the situation
Nervous	Unsure, shy, looking around, vigilant
Relaxed	Muscle tone appears soft, may move around, stand or lie down, not hectic
Submissive	Shows calming signals (such as lip licking, yawning, purposefully looking away, blinking) during interaction, may show belly, try to lick mouth, etc. (appeasement behaviours)
Restless	Jittery, moves around impatiently, may vocalise
Stressed	Uneasy, may show signs of negatively valenced arousal, e.g., wide eyes, panting, or exaggerated tail wagging
Sociable	Seeks contact/interaction, affiliative behaviour, submissiveness not included
Excited	Enthusiastic, energetic, shows signs of positive emotions

**Table 2:** List of descriptors for the QBA study on veterinary fear with correct allocations. The descriptors are listed in order of correct allocations.

Terms	Correct Allocations
Relaxed	89%
Aggressive	77%
Sociable	73%
Curious	69%
Fearful	67%
Tense	53%
Submissive	47%
Stressed	38%
Nervous	30%
Lively	22%
Restless	22%
Excited	17%

**Table 3:** Detailed allocation of descriptors in per cent.

	Aggr.	Tense	Cur.	Fearful	Lively	Nerv.	Relaxed	Subm.	Restless	Stressed	Sociable	Excited
Aggressive	77%	6%	0%	0%	0%	0%	0%	0%	0%	1%	0%	0%
Tense	**7%**	53%	2%	9%	9%	19%	2%	3%	5%	11%	1%	1%
Curious	0%	0%	69%	1%	8%	1%	0%	3%	1%	1%	**15%**	4%
Fearful	4%	**16%**	0%	67%	2%	**28%**	0%	3%	2%	8%	0%	0%
Lively	1%	1%	3%	0%	22%	0%	1%	1%	5%	1%	7%	56%
Nervous	1%	5%	1%	6%	7%	30%	1%	3%	**17%**	**17%**	0%	0%
Relaxed	0%	0%	8%	1%	4%	1%	**89%**	**22%**	1%	1%	2%	1%
Subm.	0%	2%	0%	5%	1%	3%	3%	47%	1%	1%	0%	0%
Restless	0%	2%	0%	1%	13%	6%	1%	1%	22%	8%	0%	0%
Stressed	7%	**14%**	0%	**10%**	6%	6%	2%	3%	8%	38%	0%	0%
Sociable	0%	0%	**15%**	0%	6%	1%	1%	11%	1%	1%	73%	**19%**
Excited	1%	1%	0%	1%	22%	3%	0%	3%	39%	12%	2%	17%

The descriptors on the horizontal line are the correct allocations to the definitions. Descriptors in the vertical column are those the participants allocated to the descriptor quoted above. Correct allocations are shown with a grey background, the second highest allocation is shown in **bold,** and very high wrong allocations are identified with a light grey background.

**Table 4:** Improved list of descriptor–definition pairs.

Terms	Definitions
Aggressive	Shows signs and posture of defensive or offensive aggression (staring, growling, showing teeth, snapping into the air or towards people, etc.)
Tense	Muscular tension; stiff body, may appear frozen
Curious	Interested in surroundings, looking at or approaching people or objects on its own, neutral posture, ears neutral or forward
Fearful	dog may try to flee, hide or freeze; ears back
Lively	Moving around with the legs or body but not apparently in an attempt to avoid the situation
Nervous	Unsure, shy, looking around, vigilant
Relaxed	Muscle tone appears soft, may move around, stand or lie down, not hectic
Submissive	Shows calming signals (such as lip licking, yawning, purposefully looking away, and blinking) during interaction, may show belly, try to lick mouth, etc. (appeasement behaviours)
Stressed	Uneasy, may show signs of negatively valenced arousal, e.g., wide eyes, panting, or exaggerated tail wagging
Sociable	Seeks contact/interaction, affiliative behaviour *

* The reference “submissiveness not included” for the descriptor “sociable” has been removed from this list as the descriptor–definition pairs should be used as entities and not split up in future studies.

**Table 5:** List of descriptors used by Stubsjøen et al. [23] for shelter dogs with correct allocations in per cent. The descriptors are listed according to level of correctness.

Terms	Correct Allocations
Sociable	65%
Aggressive	64%
Playful	63%
Depressed	55%
Curious	47%
Relaxed	44%
Energetic	44%
Indifferent	44%
Trustful	34%
Uncomfortable	28%
Content	27%
Restless	26%
Calming	24%
Expectant	23%
Hesitant	22%
Stressed	21%
Bored	20%
Nervous	15%
Frustrated	13%
Alert	8%

## Data Availability

Publicly available datasets were analysed in this study. These data can be found here: https://osf.io/jme5v/ (accessed on 1 August 2023).

## Appendix A

**Table A1:** List of Descriptors for Dogs in Shelter Situations [23] showing the name of the descriptor in English and Norwegian, with German translations of descriptors and German definitions. German descriptors and definitions were used in this study.

English Terms	Norwegian Terms	German Terms	German Definitions
Content	tilfreds	Zufrieden	Positive, bedürfnisstillende Aktivität, (z.B. Spiel, Zugehörigkeitsverhalten), entspannt
Uncomfortable	ukomfortabel	Unbehaglich	Ungut, depressiv, kann Schmerzen haben
Playful	leken	Verspielt	Aktiv in Spielverhalten involviert, lädt andere zum Spielen ein, fröhlich, kann Laute von sich geben und hüpfen
Depressed	nedstemt	Depressiv	Teilnahmslos, nicht an Umwelt interessiert bzw. unwillig mit Umwelt zu interagieren, gleichgültig, leerer Blick, apathisch, kann Schmerzen haben
Relaxed	avslappet	Entspannt	Keine Lautäußerung, interessiert an seiner Umgebung, nicht nervös, kann sich umherbewegen auf eine entspannte Art bzw. hinlegen, nicht depressiv
Restless	urolig	Unruhig	Ungeduldig, überspannt, bewegt sich exzessiv herum, kann Laute von sich geben, kann Aufmerksamkeit suchen. Spielen ist nicht Teil des Verhaltens
Alert	oppmerksom	Wachsam	Aufmerksam, eifrig, aktiv interessiert
Bored	Kieder seg	Gelangweilt	Inaktiv, uninteressiert, passiv
Sociable	sosial	Kontaktfreudig	Sucht Kontakt/Interaktion, freundlich, positive Interaktion mit anderen Hunden
Nervous	nervøs	Nervös	Unsicher, schüchtern, ängstlich, kann die Rute unter den Bauch einziehen, kann Laute äußern
Expectant	forventningsfull	Erwartungsvoll	Wachsam, wedelt mit der Rute, kann Laute äußern, konzentriert, kann unruhig sein
Hesitant	avventende	Zögerlich	Zurückhaltend, zurückgezogen, wachsam
Trustful	tillitsfull	Vertrauensvoll	Vertraut, anhänglich, freundlich, sucht Aufmerksamkeit
Aggressive	aggressiv	Aggressiv	Kann Laute äußern, zeigt Anzeichen und Körperhaltung von defensiver oder offensiver Aggression
Energetic	energisk	Energisch	Aktiv, kann sich lautstark äußern, beharrlich
Frustrated	frustrert	Frustriert	Konfliktverhalten, unruhig, reizbar, gestresst, kann sich lautstark äußern
Curious	nysgierrig	Neugierig	Positiv interessiert, wachsam, erkundend, aufmerksam
Calming	konfliktdempende	Beruhigend	Zeigt Beruhigungssignale (z. B. Gähnen, Lecken der Lippen/Nase, Abwenden des Kopfes, Drehen des Körpers in Richtung des anderen Hundes, Rute in niedriger Position, Ohren zurücklegen, Schnüffeln am Boden)
Indifferent	nøytral	Gleichgültig	Sucht keinen Kontakt/keine Interaktion, gibt keine Laute von sich, uninteressiert, nicht deprimiert
Stressed	stresset	Gestresst	Nervös, unruhig, kann sich wiederholendes (stereotypes) Verhalten zeigen

## Appendix B

**Table A2:** The following questions were posed in the questionnaire regarding an individual’s background. Only one answer could be chosen.

	English	German
Gender/Geschlecht	Please fill in your gender. Female. Male. Diverse. Not applicable.	Bitte geben Sie hier ihr Geschlecht an. weiblich. männlich. divers. keine Angabe.
Age/Alter	Please indicate your age. Under 20 years. Between 20 and 30 years. Between 30 and 40 years. Between 40 and 50 years. Between 50 and 60 years. Over 60 years.	Bitte geben Sie hier Ihr Alter an. Unter 20 Jahre. Zwischen 20 und 30 Jahren. Zwischen 30 und 40 Jahren. Zwischen 40 und 50 Jahren. Zwischen 50 und 60 Jahren. Über 60 Jahre.
Direct contact with dogs/Direkter Umgang mit Hunden	Please estimate how many days in the last 2 years you had direct contact with a dog/dogs. 0 days. 1 to 10 days. 10 to 50 days. 50 to 100 days. Almost daily.	An geschätzt wie vielen Tagen in den letzten 2 Jahren hatten Sie direkten Umgang mit einem Hund/Hunden? 0 Tage. 1 bis 10 Tage. 10 bis 50 Tage. 50 bis 100 Tage. nahezu täglich.
Expertise/Expertise	Indicate your expertise on the topic of dog behaviour. Uninformed. A rough idea of the meaning of dog behaviour. Good. Very good. Professional.	Wie schätzen Sie Ihre Expertise zum Thema Hundeverhalten ein? unwissend. grobe Vorstellung von der Bedeutung hündischen Verhaltens. gut. sehr gut. professionell.
Experience/Erfahrung	Please indicate your experience with dogs. I have no experience with dogs. I have little experience with dogs. I have some experience with dogs. I have already owned a dog/I am currently owning a dog. I have worked professionally with dogs/I am working professionally with dogs.	Geben Sie Ihre Erfahrung mit Hunden an. Ich habe keine Erfahrung mit Hunden. Ich habe wenig Erfahrung mit Hunden. Ich habe einige Erfahrung mit Hunden. Ich habe bereits einen Hund besessen/besitze momentan einen Hund. Ich habe beruflich mit Hunden gearbeitet/arbeite beruflich mit Hunden.
Reference of the experience/Bezug der Erfahrung	Your experience with dogs is related to what? Please choose the answer that fits the most. Loose contact with dogs. Childhood experience with a family dog. Personal responsibility for a dog in the past. Personal responsibility for a dog at the moment. Experience with dog training. Professional contact with dogs.	Ihre Erfahrung mit Hunden bezieht sich worauf? Bitte die Antwort auswählen, die am ehesten zutrifft. Loser Kontakt zu Hunden. Kindheitserfahrungen mit einem Familienhund. Persönliche Verantwortung für einen Hund in der Vergangenheit. Persönliche Verantwortung für einen Hund zum jetzigen Zeitpunkt. Erfahrung im Hundetraining. Beruflicher Kontakt zu Hunden.
Professional contact with dogs/ Beruflicher Kontakt zu Hunden	Do you work with dogs professionally? No. Yes, in a job that includes contact with dogs (e.g., vet tech, veterinary physiotherapist, animal health practitioner, or similar). Yes, as a dog trainer. Yes, as a vet specialising in behavioural therapy.	Arbeiten Sie beruflich mit Hunden? Nein Ja, in einem Beruf mit Hundebezug (z.B. TierarzthelferIn, TierphysiotherapeutIn, TierheilpraktikerIn o.Ä.) Ja, als HundetrainerIn Ja, als TierärztIn mit Zusatzbezeichnung Verhaltenstherapie

## References

[1] Fridlund A.J. (1994). Human Facial Expression: An Evolutionary View.

[2] Kraut R.E., Johnston R.E. (1979). Social and emotional messages of smiling: An ethological approach. J. Pers. Soc. Psychol..

[3] Landis C. (1924). Studies of Emotional Reactions. II. General Behavior and Facial Expression. J. Comp. Psychol..

[4] Panksepp J., Watt D. (2011). What is Basic about Basic Emotions? Lasting Lessons from Affective Neuroscience. Emot. Rev..

[5] Correia-Caeiro C., Guo K., Mills D.S. (2020). Perception of dynamic facial expressions of emotion between dogs and humans. Anim. Cogn..

[6] Bremhorst A., Mills D., Stolzlechner L., Würbel H., Riemer S. (2021). “Puppy Dog Eyes” Are Associated with Eye Movements, Not Communication. Front. Psychol..

[7] Duperrex O., Blackhall K., Burri M., Jeannot E. (2009). Education of children and adolescents for the prevention of dog bite injuries. Cochrane Database Syst. Rev..

[8] Jalongo M.R. (2008). Beyond a Pets Theme: Teaching Young Children to Interact Safely with Dogs. Early Child. Educ. J..

[9] Shen J., Rouse J., Godbole M., Wells H.L., Boppana S., Schwebel D.C. (2016). Systematic Review: Interventions to Educate Children about Dog Safety and Prevent Pediatric Dog-Bite Injuries: A Meta-Analytic Review. J. Pediatr. Psychol..

[10] Pixi Wissen, Band 14: Hunde und Wölfe: BD 14 von Imke Rudel|Buch|Zustand Gut. https://www.ebay.de/itm/133006944165.

[11] BMEL Publikationen Entdecke die Haustiere. Die kleine Tierfibel. https://www.bmel.de/SharedDocs/Downloads/DE/Broschueren/Haustierfibel.html.

[12] Reese L.A., Vertalka J.J. (2021). Understanding Dog Bites: The Important Role of Human Behavior. J. Appl. Anim. Welf. Sci..

[13] Duncan-Sutherland N., Lissaman A.C., Shepherd M., Kool B. (2022). Systematic review of dog bite prevention strategies. Inj. Prev..

[14] Philpotts I., Dillon J., Rooney N. (2019). Improving the Welfare of Companion Dogs—Is Owner Education the Solution?. Animals.

[15] Vazire S., Gosling S.D., Dickey A.S., Schapiro S.J. (2007). Measuring personality in nonhuman animals. Handbook of Research Methods in Personality Psychology.

[16] Gähwiler S., Bremhorst A., Tóth K., Riemer S. (2020). Fear expressions of dogs during New Year fireworks: A video analysis. Sci. Rep..

[17] Wemelsfelder F., Hunter T.E.A., Mendl M.T., Lawrence A.B. (2001). Assessing the ‘whole animal’: A free choice profiling approach. Anim. Behav..

[18] Pollastri I., Normando S., Contiero B., Vogt G., Gelli D., Sergi V., Stagni E., Hensman S., Mercugliano E., De Mori B. (2021). Emotional States of African Elephants (Loxodonta africana) Kept for Animal–Visitor Interactions, as Perceived by People Differing in Age and Knowledge of the Species. Animals.

[19] Arena L., Wemelsfelder F., Messori S., Ferri N., Barnard S. (2019). Development of a fixed list of terms for the Qualitative Behavioural Assessment of shelter dogs. PLoS ONE.

[20] Clarke T., Pluske J.R., Fleming P.A. (2016). Are observer ratings influenced by prescription? A comparison of Free Choice Profiling and Fixed List methods of Qualitative Behavioural Assessment. Appl. Anim. Behav. Sci..

[21] Ceballos M.C., Góis K.C.R., Sant’Anna A.C., Wemelsfelder F., Paranhos da Costa M. (2021). Reliability of qualitative behavior assessment (QBA) versus methods with predefined behavioral categories to evaluate maternal protective behavior in dairy cows. Appl. Anim. Behav. Sci..

[22] Bokkers E., de Vries M., Antonissen I., de Boer I. (2012). Inter- and intra-observer reliability of experienced and inexperienced observers for the Qualitative Behaviour Assessment in dairy cattle. Anim. Welf..

[23] Stubsjøen S.M., Moe R.O., Bruland K., Lien T., Muri K. (2020). Reliability of observer ratings: Qualitative behaviour assessments of shelter dogs using a fixed list of descriptors. Vet. Anim. Sci..

[24] Cooke A.S., Mullan S.M., Morten C., Hockenhull J., Lee M.R.F., Cardenas L.M., Rivero M.J. (2022). V-QBA vs. QBA—How Do Video and Live Analysis Compare for Qualitative Behaviour Assessment?. Front. Vet. Sci..

[25] Brscic M., Wemelsfelder F., Tessitore E., Gottardo F., Cozzi G., Van Reenen C. (2009). Welfare assessment: Correlations and integration between a Qualitative Behavioural Assessment and a clinical/ health protocol applied in veal calves farms. Ital. J. Anim. Sci..

[26] Dalla Costa E., Dai F., Murray L.M.A., Cannas S., Canali E., Zanella A.J., Minero M. (2021). The development of the AWIN welfare assessment protocol for donkeys. Braz. J. Vet. Res. Anim. Sci..

[27] Tami G., Gallagher A. (2009). Description of the behaviour of domestic dog (*Canis familiaris*) by experienced and inexperienced people. Appl. Anim. Behav. Sci..

[28] López Arroyo B., Roberts R.P. (2020). What wine descriptors really mean A comparison between dictionary definitions and real use. J. Wine Res..

[29] Moss R., Healey K., Hayward L., McSweeney M.B. (2021). Projective mapping and ultra-flash profile studies should include a list of descriptors and definitions: An investigation into descriptors used by untrained panelists. J. Sens. Stud..

[30] Li J., Predic M., Gómez M.I. (2020). The Effect of Subjective and Objective Tasting Sheet Descriptors on Tasting Room Sales in New York State. Cornell Hosp. Q..

[31] Wansink B., van Ittersum K., Painter J.E. (2005). How descriptive food names bias sensory perceptions in restaurants. Food Qual. Prefer..

[32] Rast P. (2011). Verbal knowledge, working memory, and processing speed as predictors of verbal learning in older adults. Dev. Psychol..

[33] Phythian C., Michalopoulou E., Duncan J., Wemelsfelder F. (2013). Inter-observer reliability of Qualitative Behavioural Assessments of sheep. Appl. Anim. Behav. Sci..

[34] Wan M., Bolger N., Champagne F.A. (2012). Human Perception of Fear in Dogs Varies According to Experience with Dogs. PLoS ONE.

[35] Kujala M.V., Somppi S., Jokela M., Vainio O., Parkkonen L. (2017). Human Empathy, Personality and Experience Affect the Emotion Ratings of Dog and Human Facial Expressions. PLoS ONE.

[36] Root-Gutteridge H., Brown L.P., Forman J., Korzeniowska A.T., Simner J., Reby D. (2021). Using a new video rating tool to crowd-source analysis of behavioural reaction to stimuli. Anim. Cogn..

[37] Andreasen S.N., Wemelsfelder F., Sandøe P., Forkman B. (2013). The correlation of Qualitative Behavior Assessments with Welfare Quality® protocol outcomes in on-farm welfare assessment of dairy cattle. Appl. Anim. Behav. Sci..

[38] Battini M., Barbieri S., Vieira A., Can E., Stilwell G., Mattiello S. (2018). The use of qualitative behaviour assessment for the on-farm welfare assessment of dairy goats. Animals.

[39] Grosso L., Battini M., Wemelsfelder F., Barbieri S., Minero M., Dalla Costa E., Mattiello S. (2016). On-farm Qualitative Behaviour Assessment of dairy goats in different housing conditions. Appl. Anim. Behav. Sci..

[40] Napolitano F., De Rosa G., Serrapica M., Braghieri A. (2015). A continuous recording approach to qualitative behaviour assessment in dairy buffaloes (*Bubalus bubalis*). Appl. Anim. Behav. Sci..

[41] Rutherford K.M.D., Donald R.D., Lawrence A.B., Wemelsfelder F. (2012). Qualitative Behavioural Assessment of emotionality in pigs. Appl. Anim. Behav. Sci..

[42] Serrapica M., Boivin X., Coulon M., Braghieri A., Napolitano F. (2017). Positive perception of human stroking by lambs: Qualitative behaviour assessment confirms previous interpretation of quantitative data. Appl. Anim. Behav. Sci..

[43] Delfour F., Monreal-Pawlowsky T., Vaicekauskaite R., Pilenga C., Garcia-Parraga D., Rödel H.G., García Caro N., Perlado Campos E., Mercera B. (2020). Dolphin Welfare Assessment under Professional Care: ‘Willingness to Participate’, an Indicator Significantly Associated with Six Potential ‘Alerting Factors’. J. Zool. Bot. Gard..

[44] Patel F., Wemelsfelder F., Ward S.J. (2019). Using Qualitative Behaviour Assessment to Investigate Human-Animal Relationships in Zoo-Housed Giraffes (*Giraffa camelopardalis*). Animals.

[45] Rose P., Riley L. (2019). The use of Qualitative Behavioural Assessment to zoo welfare measurement and animal husbandry change. J. Zoo Aquar. Res..

[46] Menchetti L., Righi C., Guelfi G., Enas C., Moscati L., Mancini S., Diverio S. (2019). Multi-Operator Qualitative Behavioural Assessment for dogs entering the shelter. Appl. Anim. Behav. Sci..

[47] Walker J.K., Dale A.R., D’Eath R.B., Wemelsfelder F. (2016). Qualitative Behaviour Assessment of dogs in the shelter and home environment and relationship with quantitative behaviour assessment and physiological responses. Appl. Anim. Behav. Sci..

[48] Wemelsfelder F. (2007). How animals communicate quality of life: The qualitative assessment of behavior. Anim. Welf..

[49] Battini M., Stilwell G., Vieira A., Barbieri S., Canali E., Mattiello S. (2015). On-FarmWelfare Assessment Protocol for Adult Dairy Goats in Intensive Production Systems. Animals.

[50] Dalla Costa E., Dai F., Lebelt D., Scholz P., Barbieri S., Canali E., Zanella A., Minero M. (2016). Welfare assessment of horses: The AWIN approach. Anim. Welf..

[51] Winckler C. (2014). On-farm animal welfare assessment and welfare improvement in dairy cattle. AgroLife Sci. J..

[52] King T., Flint H.E., Hunt A.B.G., Werzowa W.T., Logan D.W. (2022). Effect of Music on Stress Parameters in Dogs during a Mock Veterinary Visit. Animals.

[53] Tuyttens F.A.M., de Graaf S., Heerkens J.L.T., Jacobs L., Nalon E., Ott S., Stadig L., Van Laer E., Ampe B. (2014). Observer bias in animal behaviour research: Can we believe what we score, if we score what we believe?. Anim. Behav..

[54] Burghardt G.M., Bartmess-LeVasseur J.N., Browning S.A., Morrison K.E., Stec C.L., Zachau C.E., Freeberg T.M. (2012). Perspectives—Minimizing Observer Bias in Behavioral Studies: A Review and Recommendations: Minimizing Observer Bias in Behavioral Research. Ethology.

[55] Wemelsfelder F., Hunter E.A., Mendl M.T., Lawrence A.B. (2000). The spontaneous qualitative assessment of behavioural expressions in pigs: First explorations of a novel methodology for integrative animal welfare measurement. Appl. Anim. Behav. Sci..

[56] Meagher R.K. (2009). Observer ratings: Validity and value as a tool for animal welfare research. Appl. Anim. Behav. Sci..

[57] Hintze S., Murphy E., Bachmann I., Wemelsfelder F., Würbel H. (2017). Qualitative Behaviour Assessment of horses exposed to short-term emotional treatments. Appl. Anim. Behav. Sci..

[58] Wess L., Böhm A., Schützinger M., Riemer S., Yee J.R., Affenzeller N., Arhant C. (2022). Effect of cooperative care training on physiological parameters and compliance in dogs undergoing a veterinary examination—A pilot study. Appl. Anim. Behav. Sci..

[59] Unipark—Das Akademische Programm von Tivian|Impressum. https://www.unipark.com/impressum/.

[60] Brscic M., Dam Otten N., Contiero B., Kirchner M.K. (2019). Investigation of a Standardized Qualitative Behaviour Assessment and Exploration of Potential Influencing Factors on the Emotional State of Dairy Calves. Animals.

[61] Zhao F., Schnotz W., Wagner I., Gaschler R. (2020). Texts and pictures serve different functions in conjoint mental model construction and adaptation. Mem. Cognit..

[62] Pollak S.D., Sinha P. (2002). Effects of early experience on children’s recognition of facial displays of emotion. Dev. Psychol..

[63] Pollak S.D., Kistler D.J. (2002). Early experience is associated with the development of categorical representations for facial expressions of emotion. Proc. Natl. Acad. Sci. USA.

[64] Sullivan M.W., Bennett D.S., Carpenter K., Lewis M. (2008). Emotion Knowledge in Young Neglected Children. Child Maltreat..

[65] Lakestani N.N., Donaldson M.L., Waran N. (2014). Interpretation of Dog Behavior by Children and Young Adults. Anthrozoös.

[66] Sullivan S.K., Kim A., Vinicius Castilho L., Harris L.T. (2022). Comparing emotion inferences from dogs (*Canis familiaris*), panins (*Pan troglodytes*/*Pan paniscus*), and humans (*Homo sapiens*) facial displays. Sci. Rep..

[67] Riggio G., Gazzano A., Zsilák B., Carlone B., Mariti C. (2020). Quantitative Behavioral Analysis and Qualitative Classification of Attachment Styles in Domestic Dogs: Are Dogs with a Secure and an Insecure-Avoidant Attachment Different?. Animals.

[68] Koo T.K., Li M.Y. (2016). A Guideline of Selecting and Reporting Intraclass Correlation Coefficients for Reliability Research. J. Chiropr. Med..

[69] Lucy J.A. (2001). Sapir–Whorf Hypothesis. International Encyclopedia of the Social & Behavioral Sciences.

[70] Perlovsky L. (2009). Language and emotions: Emotional Sapir–Whorf hypothesis. Neural Netw..

[71] Sung Y., Chang K., Chang T., Yu W. (2010). How many heads are better than one? The reliability and validity of teenagers’ self- and peer assessments. J. Adolesc..

[72] Jaramillo F.M., Oliveira T.M., Silva P.E.A., Trindade P.H.E., Baccarin R.Y.A. (2023). Development of a fixed list of descriptors for the qualitative behavioral assessment of thoroughbred horses in the racing environment. Front. Vet. Sci..

[73] Riemer S., Heritier C., Windschnurer I., Arhant C., Pratsch L., Affenzeller N. (2021). A Review on Mitigating Fear and Aggression in Dogs and Cats in a Veterinary Setting. Animals.

